# Serum IgE Reactivity Profiling in an Asthma Affected Cohort

**DOI:** 10.1371/journal.pone.0022319

**Published:** 2011-08-04

**Authors:** Tania Dottorini, Gabriella Sole, Luisa Nunziangeli, Francesca Baldracchini, Nicola Senin, Giorgio Mazzoleni, Carla Proietti, Lenuta Balaci, Andrea Crisanti

**Affiliations:** 1 Department of Experimental Medicine and Biochemical Sciences, University of Perugia, Perugia, Italy; 2 National Research Council of Italy (CNR), Institute of Genetics and Biomedical Research (IRGB), Cagliari, Italy; 3 Microtest Matrices LtD, London, United Kingdom; 4 Department of Industrial Engineering, University of Perugia, Perugia, Italy; 5 Biological Sciences, Imperial College London, London, United Kingdom; University of Pittsburgh, United States of America

## Abstract

**Background:**

Epidemiological evidence indicates that atopic asthma correlates with high serum IgE levels though the contribution of allergen specific IgE to the pathogenesis and the severity of the disease is still unclear.

**Methods:**

We developed a microarray immunoassay containing 103 allergens to study the IgE reactivity profiles of 485 asthmatic and 342 non-asthmatic individuals belonging to families whose members have a documented history of asthma and atopy. We employed *k-means* clustering, to investigate whether a particular IgE reactivity profile correlated with asthma and other atopic conditions such as rhinitis, conjunctivitis and eczema.

**Results:**

Both case-control and parent-to-siblings analyses demonstrated that while the presence of specific IgE against individual allergens correlated poorly with pathological conditions, particular reactivity profiles were significantly associated with asthma (p<10E-09). An artificial neural network (ANN)-based algorithm, calibrated with the profile reactivity data, correctly classified as asthmatic or non-asthmatic 78% of the individual examined. Multivariate statistical analysis demonstrated that the familiar relationships of the study population did not affect the observed correlations.

**Conclusions:**

These findings indicate that asthma is a higher-order phenomenon related to patterns of IgE reactivity rather than to single antibody reactions. This notion sheds new light on the pathogenesis of the disease and can be readily employed to distinguish asthmatic and non-asthmatic individuals on the basis of their serum reactivity profile.

## Introduction

Asthma is one of the most common diseases affecting both adults and children and accounts for up to 300 million [1] cases worldwide. Worryingly, its frequency has increased annually during the last five decades [2], [3]. Both genetic (cytokines and immune response genes) [4], [5], developmental and environmental factors (viral infections [6], allergens [7] and occupational exposures [8] have been associated with asthma susceptibility, age of onset and severity. Although the pathogenesis of the disease has not been fully elucidated yet, a major risk factor is the development of immune responses to foreign antigens, that are characterized by the production of antigen-specific IgE [9]. This notion has been first inferred from observations showing that the prevalence of asthma was closely related to the serum IgE level standardized for age and sex [10]. Overwhelming evidence has confirmed the role of IgE in atopic asthma, while several studies have also revealed a link between IgE and non-atopic asthma [11]. More controversial is the role of antigen specific IgE in determining the onset and severity of the disease. Several studies have unraveled strong relationships among exposure to house dust mite (HDM), the presence of serum IgE directed against the mite allergens, and asthma [12]. However, a large number of individuals worldwide, particularly those living in some regions of USA and Scandinavia, have low lifetime exposure to mite antigens, but do not show any decrease in the prevalence and the severity of asthma [13]. Therefore, other antigens -either alone or in combination- ought to have the ability to elicit an IgE response and play a role in the pathogenesis of the disease. Indeed the links among antigen exposure, IgE production, and occurrence and/or severity of asthma seem to involve an unexpected number of factors, and a nonlinear relationship between exposure and response appears to exist [14]. To date, studies of the association between specific IgE and asthma have focused on analyzing either one or a few antigens at a time, like for example those describing the role of HDM [15]–[18]. The disproportion between the repertoire of known allergens and the number of antigens that have been analyzed may well explain the difficulties encountered in establishing the role of specific IgE in the pathogenesis of asthma.

We generated a microarray containing a vast repertoire of allergens (103) that forms the substrate of an antibody-capture assay to investigate the IgE reactivity profiles of 872 individuals belonging to families with documented history and diagnosis of asthma and atopic diseases. Then, we searched for associations between IgE reactivity profiles and atopic diseases including asthma, rhinitis, conjunctivitis and eczema in a case-control and parent-to-siblings study. Multivariate analysis was carried out to assess the effect of family relationships on the statistical analysis. The results of the IgE reactivity profiles were utilized to develop and validate an artificial neuronal network classifier capable of distinguishing asthmatic and non-asthmatic individuals with high accuracy.

## Materials and Methods

### Population case study

The sample consisted of a total of 872 sera, including 442 parents and their progeny (430 individuals) (Table 1). Within the study group, 428 children and 57 parents (55.62% of the total) were diagnosed with asthma, 342 parents (39.22% of the total) were classified as non asthmatic, though some of them suffered from atopy related disorders such as rhinitis, conjunctivitis and eczema, a remaining 5.16% were classified as undefined asthma diagnosis. Atopic asthmatic sibling pairs (sibs) and trios were collected over a period of 4 years, mainly from pediatric and pneumological centers. All patients were of Sardinian origin for at least 3 generations and their age at visit was above 6 years to avoid subjects with transient symptoms. At the recruitment sessions, each subject was interviewed, disease status ascertained by physical examination, permission asked to access personal health records, and blood samples were collected. Each participant signed an informed consent form approved by the local ethics committee (Azienda Sanitaria Locale number 8 protocol 24/Comitato Etico/02, authorization number 4737). Asthma was diagnosed by a pulmonary physician, in accordance with the American Thoracic Society criteria [19]. Pulmonary function was evaluated by spirometry. A physician administered a questionnaire collecting clinical history and classifying asthma severity in four levels according to the World Health Organization guidelines (Global Initiative for Asthma). The use of asthma drugs and any other medication was recorded. Atopy was detected by positive skin testing to common inhalant allergens by standard methods. Patients with history of early asthma onset were interviewed by a physician about persistency of asthma symptoms after the completion of puberty (18 years).

**Table 1:** The population study samples.

	Offspring	Parents	Parent and offspring
	(N = 430)	(N = 442)	(N = 872)
**Age**			
Minimum	6.00	25.00	6.00
Maximum	65.80	88.60	88.60
Mean (S.D.)	21.31 (12.02)	47.05 (10.47)	34.30 (17.10)
Median	20.00	46.00	36.00
**Sex**			
Male (%)	245 (56.97*)	201 (45.47)	446 (51.15)
Female (%)	185 (43.03)	241 (54.53)	426 (48.85)
**Rhinitis**			
Affected (%)	324 (75.35)	104 (23.53)	428 (49.08)
Not affected (%)	92 (21.39)	310 (70.14)	402 (46.10)
Undefined diagnosis (%)	14 (3.26)	28 (6.33)	42 (4.82)
**Eczema**			
Affected (%)	132 (30.70)	40 (9.05)	172 (19.72)
Not affected (%)	281 (65.35)	365 (82.58)	646 (74.08)
Undefined diagnosis (%)	17 (3.95)	37 (8.37)	54 (6.20)
**Conjuntivitis**			
Affected (%)	191 (44.42)	74 (16.74)	265 (30.39)
Not affected (%)	201 (46.74)	302 (68.33)	503 (57.68)
Undefined diagnosis (%)	38 (8.83)	66 (14.93)	104 (11.93)
**Asthma**			
Affected (%)	428 (99.50)	57 (12.90)	485 (55.62)
Not affected (%)	0	342 (77.37)	342 (39.22)
Undefined diagnosis (%)	2 (0.50)	43 (9.73)	45 (5.16)
**Age of asthma onset**			
Minimum	1.00	-	-
Maximum	57.00	-	-
Mean (S.D.)	10.35 (10.82)	-	-
**Asthma severity**			
1° level (%)	115 (26.74)	-	-
2° level (%)	76 (17.67)	-	-
3° level (%)	75 (17.44)	-	-
4° level (%)	132 (30.69)	-	-
Undefined diagnosis (%)	32 (7.44)	-	-
**Asthma persistency** *			
Persistent (%)	290 (67.44)	-	-
Not persistent (%)	35 (8.14)	-	-
Undefined diagnosis (%)	105 (24.42)	-	-

*Persistency of asthma symptoms after the age of 18.

### Development of IgE microarray immunoassay

The serum IgE reactivity was analyzed using a fluorescence immunoassay that incorporates as a substratum a microarray of 103 allergens (Table S1) including 95 extracts and 8 recombinant proteins representative of 11 distinct allergen classes chosen amongst those most frequently associated with atopic diseases in Southern-Central Europe. Comparisons performed between the ELISA and the microarray assay for 6 common allergens, revealed that the overall diagnostic performance, as defined by clinical sensitivity and specificity, of the microarray immunoassay was very good [20]. To generate the array the allergens were printed onto aldehyde-activated glass microscope slides in duplicates at randomised positions in the array. The immunoassay procedure consisted of four phases (*printing*, *processing, scanning, quantification and analysis*). Two chips of 103 allergens each, were printed onto each slide using high–speed robotics (Microgrid Compact; Biorobotics). Allergens (Allergopharma) were spotted onto the arrays in the following spotting buffers: PBS pH 7.4, glycine pH 2.4, Borate pH 9.4, glycerol 10%, DTT 5 mM, SDS (0.2%; 0.05%), Tween 20 (0.01%) at a spotting concentration ranging from 0.008 to 3 mg/ml. Bound IgE were revealed incubating the slides first with serum sample (100 µl- 60 minutes at 37°C), followed by a secondary mouse monoclonal antibody directed against human IgE (100 µl- 45 minutes at 37°C), followed by anti-mouse IgG HRP conjugated antibody (100 µl- 45 minutes at 37°C) and finally incubated with tyramide-Alexa 555 (100 µl- 15 minutes at 37°C).

The ScanArray™ software provided by Perkin Elmer Life Sciences Inc. was used to scan the slides and to acquire the fluorescence. After subtracting the background signal the concentrations (IU/ml) of allergen-bound IgE were determined by interpolating the signal with an internal calibration curve printed onto each microarray. To assign IU/ml values to the calibration curve, we used an external Reference Curve generated by microarray slides printed with replicates of Goat anti-Human IgE and incubated with increasing concentrations of human IgE (WHO Reference standard 0.35, 1.0, 3.5, 10.0, 50.0 IU/ml). The signal collected from the allergens was interpolated with the calibration curve to obtain the IU/ml value, and translated into a Class Score by plotting the data in a standard reactivity scale. Class Score values: (CLASS 0 (less than 0.35 IU/ml); CLASS 1 (0.35–0.7 IU/ml); CLASS 2 (0.71–3.5 IU/ml); CLASS 3 (3.51–17.5 IU/ml); CLASS 4 (17.51–50 IU/ml); CLASS 5 (50.01–100 IU/ml).

The microarray data has been submitted to Gene Expression Omnibus - GEO-NCBI, accession numbers GSE20020; Platform GPL9968 “Allergochip for asthma diagnosis”

A detailed description of the array procedure is described in the supporting information (Text S1).

### Statistical analysis

Reactivity data from each serum were quantified in IU/ml, transformed into class score and encoded with 103-dimensional vectors (1 dimension for each allergen) using *Cluster 3.0* [21] to generate individual profiles. We used *k*-means clustering to group sera into distinct clusters on the basis of similarities in their reactivity profiles, while *MapleTree* [22] was used for visualizing the clustering results. Clustering is a statistical technique for collecting objects into a fixed number of groups (clusters) so that each group contains only similar objects. In this work we used k-means clustering, a clustering technique where the target number of clusters (k) is user-defined. In clustering, similarity is determined by attributes associated to each object. In this case, each object is a serum, and its attributes are the reactivity data derived from 103 allergens; k-means clustering was therefore aimed at grouping 872 sera into k clusters, so that each cluster would contain only sera with similar reactivity data (derived from their 103 allergens). To assess whether two objects (sera) are similar, which is needed to collect them in the same cluster, their attributes (103 allergen reactivity values) must be compared: this is achieved by defining a *similarity metric*, i.e. a mathematical formula that combines attribute values of two objects into a meaningful measure of their similarity. In this work, *Euclidean distance* was adopted as similarity metric, i.e. the square root of the sum of the squared differences of each attribute. The smaller the Euclidean distance, the more similar the two objects. The k-means clustering algorithm works by iteratively inserting each object (serum) into a tentative cluster, and by refining the insertion process until the similarity of the objects within each cluster is maximised. In this case, convergence to an optimal solution was achieved within 10,000 iterations of the clustering algorithm. The disadvantage of k-means clustering is that the number of clusters (k) is user defined. Therefore, to determine what value of k would produce optimal results, we first ran multiple clustering analyses with different k values (from 1 to 14 and 20) and then we analysed the clustering results using indicators that measure how similar the objects are within each cluster, and how different the clusters are with each other. We used five performance indicators (Silhouette index, Dunn index, Davies Bouldin, C-index and Isolation index), as provided by the Machaon software [23], to validate the statistical significance of each partitioning attempt; we determined that K = 3 was the most significant result overall.

Then, we proceeded to determine whether the clustering results would contain significant information also concerning associations with age, sex, and the presence of a pathological condition (asthma, rhinitis, etc.), its persistency and/or severity, age at onset, etc. Statistical tests such as Pearson's χ2 and Kruskall-Wallis non-parametric test were run within the SPSS software and Excel to investigate whether the frequency of a pathological condition differed significantly in the clusters and whether each cluster significantly differed from the study population taken as a whole. In particular, the χ2 test was used for analysing binary attributes (i.e. asthmatic vs. non-asthmatic), while the Kruskall-Wallis test was performed on discrete numeric variables (such as the age of asthma onset).

### Artificial neural network asthma classifier

An artificial neural network (ANN), known as the *radial basis function* (RBF) [24] was adopted to implement the asthma classifier. Professional software applications, which have modules specifically dedicated for ANN, such as the RBF (Radial Basis Function Algorithm - SPSS 17.0.) were utilized for developing the classifier. The neural network analysis was accomplished by using as input data, a sample data set that includes 51 allergens and the sera reactivity profiles of 827 individuals. Within the sample, 485 are asthma positive individuals, and 342 negative. To evaluate the actual improvement that was achieved by reducing the number of allergens to be considered by the classifier (by means of the Mann-Whitney test, as illustrated previously), a separate RBF was trained on the complete set of allergens, and then its results compared with the RBF operating on the filtered set. The sample was first randomized and the size of the training sample used was about 60% of the entire population, while the remaining individuals were left for validation purposes (testing 10% and holdout of 30%). The training subset was selected using randomization criterion that ensures the representativeness of the sample with respect to the entire population. This is to ensure that the RBF replicates a behaviour that is representative for the whole population. The whole supervised training process was repeated 10 times, each time on a new, previously untrained network, and each time with a new randomized subset of the original population. This is to observe any variation in performance, which may be linked to variability in the representativeness of the training sample. There are three layers in the RBF network (Input, RBF and output layer). There are many types of radial basis functions; we used the Normalized RBF (NRBF). To have an estimate of the real efficiency of the neural network, the neural network has been run 10 different times. And the overall efficiency of the Network is given as the mean of the 10 different trials.

### Generalized Estimating Equations (GEE) analysis

The results were processed with Generalized Estimating Equations (GEEs) [25] to test for associations between asthma and IgE reactivity taking into account for familiar relationships between asthmatic and non-asthmatic individuals. GEEs are a semi-parametric regression technique useful for fitting the parameters of a model where unknown correlation between variables may be present. The GEE probit analysis is similar to probit regression models (appropriate for dichotomous dependent variable and a set of explanatory variables), but it also takes into account relationships amongst members of the same cluster, in this case parent to sibling relationships. In this study, a “working” correlation matrix for the clusters, which models the dependence of each observation with other observations in the same cluster, was specified. The familial correlation was modelled assuming exchangeable correlation for the working correlation matrix, i.e. all measurements on the same cluster are equally correlated. Regardless of the specification of the correlation, GEE models are robust to the misspecification of the correlations structure. Additionally, we selected robust standard errors (sandwich estimators as opposed to conventional standard errors) that allowed the estimates to be valid even in the event of misspecification of the correlation structure.

The association between asthma and specific IgE against single allergens was first studied by GEE univariate analyses, determining possible significant explanatory variables to be included in the model runs. Multicollinearity among potential explanatory variables was investigated using regression diagnostic capabilities to ensure no model-burdening correlations exist between variables. In these analyses specific IgE against single allergens were regarded as factors with 4 levels, since class scores 5 having low frequencies were unified to class score 4. Finally, in order to identify variables predisposing independently to asthma, we performed a multivariate logistic regression model for those variables found from the univariate analysis to have *P*<0.05, using a forward modelling strategy and the corresponding odds ratio, confidence interval and statistical significance were calculated for each class score of specific IgE in the model. Age, gender and the interaction between these variables were included as covariates. The GEE was run in SPSS v16.0.

## Results

**Figure 1:**
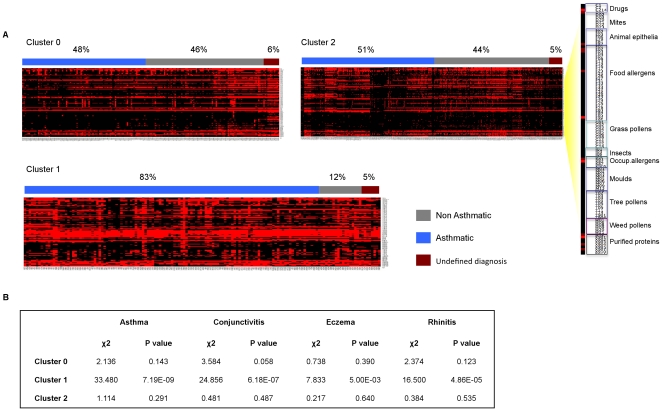
K-means clustering of serum reactivity profiles of 872 sera (columns) against the arrayed 103 allergens (rows). (A) The serum reactivity profile generated for each cluster (0–2) at *k* = 3 are visualized. Each column represents an individual serum grouped as asthmatic (blue), non-asthmatic (gray) and undefined diagnosis (dark red). Reactivity values to individual allergens are classified as either positive (scale of red - class score 1–5) and negative (black - class score 0). (B) The distribution of atopic conditions in each cluster was assessed using the Pearson's *X* ^2^ test.

We have investigated the IgE serum reactivity of 872 individuals belonging to 283 Sardinian families in which all the progeny (1 to 3 siblings) was affected by atopic asthma (Table 1). The individuals enrolled in this study included the two parents and their siblings, mostly children below the age of 27 (75%). The rationale of using this particular cohort originates from the notion that asthma has both genetic and environmental components that are not well defined yet. A sample study comprised of families (parents-children) from a genetically isolated population like the Sardinians offers a unique opportunity to minimize possible sources of genetic heterogeneity and to reduce difference in allergen exposure. The immunoassay was calibrated (using an internal standard curve) to measure the amount of specific IgE binding to each of the arrayed antigens (Table S1) ranging from 0.35 IU/ml to 100 UI/ml. The reactivity values in UI/ml were converted into class scores using a validated 0–5 scale [20]. This approach generated 872 distinct IgE reactivity profiles and an excess of 90,000 antibody-antigen determinations. A color-coded digital profile (from black to increasing intensity of red) matching the IgE class score (0 to 5) against the arrayed allergens was generated for each serum (Figure 1).

**Figure 2:**
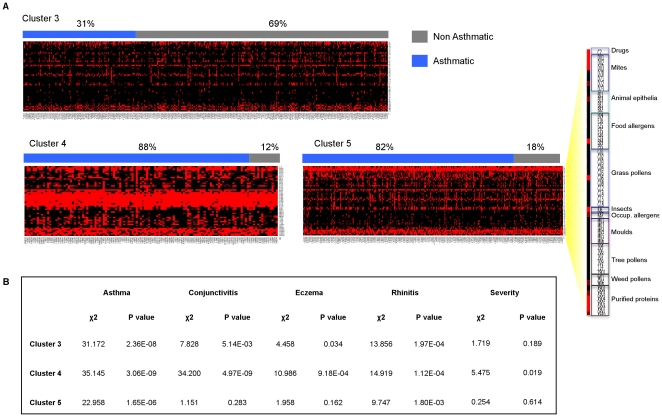
K-means clustering of serum reactivity profiles of 827 sera (columns) against the subset of asthma relevant allergens (rows). (A) The serum reactivity profile generated for each cluster (3–5) at *k* = 3 are visualized. Each column represents an individual serum grouped as asthmatic (blue) and non-asthmatic (gray). Reactivity values to individual allergens are classified as either positive (scale of red - class score 1–5) and negative (black - class score 0). (B) The distribution of atopic conditions in each cluster was assessed using the Pearson's *X* ^2^ test.

We employed *k*-means clustering [26], a partitioning method commonly used to identify group structure within microarray data to investigate the structure of the reactivity profiles. A significant amount of literature work addresses clustering as a valid statistical method to study asthma [15]. The clustering algorithm was run with different values of *k* (from 3 to 14 and 20) to split the profiles into different groups. Statistical analysis computed with Machaon software [22], showed that *k* = 3 is the partition value that, by arranging the profiles into three clusters, maximizes intra cluster similarities and inter cluster differences (Table S2). To validate the capability of the clustering analysis to separate asthmatic and non-asthmatic reactivity profiles, we assessed the frequency of asthmatic and non-asthmatic individuals in the three clusters in a sample consisting of 114 individuals chosen amongst the parents equally divided between asthmatic (cases) and non-asthmatic (controls) individuals. Furthermore, cases and controls were selected so that matched pairs could be formed in terms of age and sex (Table S3). This analysis showed that the distribution of cases and controls significantly differed in two out of three clusters. While cluster 2 did not show differences in the number of cases and controls, both cluster 0 and 1 were enriched with non-asthmatic and asthmatic individuals respectively (p<0.01 in the Pearson's χ^2^) thus indicating that cases and controls had distinct reactivity profiles against the arrayed allergens (Table S4). We investigated how members of the entire 283 parent-siblings cohort pairs were distributed in cluster 0, 1 and 2 stratified according to the presence of atopic diseases such asthma, conjunctivitis, eczema, rhinitis and other traits (age, sex, disease persistency and severity). This analysis indicated that the three clusters were significantly different in terms of frequency of asthma (p = 2.95E-12), conjunctivitis (p = 6.81E-10), eczema (p = 1.10E-03), rhinitis (p = 2.35E-07) and sex (Table 2). In agreement with the case-control analysis, cluster 1 showed an impressively higher proportion of asthmatic individuals (83%), if compared to the other two clusters (Figure 1A), and also with respect to the entire study sample (χ^2^ = 33.480, p = 7.19E-09) (Figure 1B). Similarly significant associations could also be observed for conjunctivitis and rhinitis with cluster 1. The partitioning of the profiles highlighted also an unequal distribution of the familiar nuclei (Table S5). While cluster 1 contained a significantly high proportion of affected children, but very few parents, clusters 0 and 2 were enriched for members of the same families and showed a similar percentage of asthmatic and non-asthmatic individuals. We reasoned that family members segregating in these two clusters showed a similar IgE recognition profile irrespectively of asthma, possibly because of the common exposure to particular sets of the arrayed allergens. The array was designed without a detailed knowledge of the exposure to allergens of the study population and without any a priori assumption on the role of particular allergens in eliciting an IgE response associated with asthma. It is therefore not surprising that some allergens are rarely recognized while others show similar percentages of reactivity in asthmatic and non-asthmatic individuals. The reactivity against these allergens contributes to the formation of the profile and could represent a source of “background noise” that masks relevant associations with the asthma status. To address this problem we attempted to generate new profiles using only the allergens that individually showed some differences in the IgE reactivity between asthmatic and non-asthmatic individuals using the Mann-Whitney U test at a threshold of p<0.05. This analysis generated a list of 51 relevant allergens (Table S6) that was employed to generate new profiles and perform clustering association analysis at *k* = 3. The new clusters (cluster 3, 4 and 5) revealed a striking increase in the statistical significance of the distribution of asthma amongst the clusters when examining the case control groups (p = 3.26E-3) (data not shown) and the entire parent-siblings cohort (p = 3.37E-52) as well as in the other examined atopic conditions (Table S7). Cluster 4 showed high similarity to cluster 1 in terms of both profile structure and composition containing a significantly high proportion of asthmatic sera (χ^2^ = 35.145, p = 3.06E-09) (Figure 2). The other two clusters differed substantially from those generated with the complete set of allergens. Cluster 5 was significantly enriched with the reactivity profiles of most of the asthmatic individuals not included in cluster 4 (χ^2^ = 22.958, p = 1.65E-06), whereas cluster 3 contained most of the non-asthmatic individuals (χ^2^ = 31.172, p = 2.36E-08). A very strong association could also be observed in cluster 4 with both conjunctivitis, and rhinitis compared to the other clusters. Notably, the clusters generated with the filtered set of allergens did not show a significant co-segregation of family members (Table S8). Cluster 4 and 5 (containing nearly all the asthmatic individuals) shared some common features in their IgE reactivity profile (Figure S1). Twenty out of the 51 allergens (mainly inhalant) were recognized by the sera of the two clusters but those of cluster 4 also reacted against nine allergens mainly derived from the food and grass (allergen 19–23 and 27–30 of Table S6). Notably, cluster 4 showed a higher proportion of individuals with diagnosis of severe asthma (severity class 3 and 4) compared to all other clusters (Table S7) and to the population study sample (Figure 2B).

**Table 2:** Distribution of atopic traits amongst the reactivity profiles.

allergens = 103*	Asthma (%)			Conjunctvitis (%)		Eczema (%)		Rhinitis (%)		Sex (%)		Persistency (%)		Severity (%)†		Age onset‡
	−	+	u.d§	−	+	−	+	−	+	M	F	−	+	1	2	
**Cluster 0**	45.9	48.5	5.6	74.5	25.5	82.5	17.5	56.1	43.9	52.6	47.4	15.1	84.9	53.6	46.4	4.0
**Cluster 1**	12.2	82.6	5.2	41.8	58.2	67.6	32.4	28.1	71.9	62.8	37.2	11.1	88.9	38.5	61.5	8.5
**Cluster 2**	44.3	50.8	4.9	68.8	31.2	80.9	19.1	51.5	48.5	47.5	52.5	9.6	90.4	50.2	49.8	7.0
**Total**	39.2	55.6	5.2	65.5	34.5	79.0	21.0	48.4	51.6	51.3	48.7	10.9	89.1	47.9	52.1	
**χ2**	56.719			42.215		13.591		30.524		11.000		1.276		5.260		18.930
**p-value**	2.95E-12			6.81E-10		1.10E-03		2.35E-07		4.1E-03		0.528		0.072		7.75E-05

*Number of allergens utilized to generate the profiles of clusters 0–2.

† Asthma severity was classified by a physician in four levels according to the World Health Organization guidelines (Global Initiative for Asthma). For simplicity we considered individuals being of level 1 to 2 as one group (column 1) and individuals with higher severity, level from 3 to 4, as one group (column 2).

‡ Median values.

§ Undefined diagnosis.

**Figure 3:**
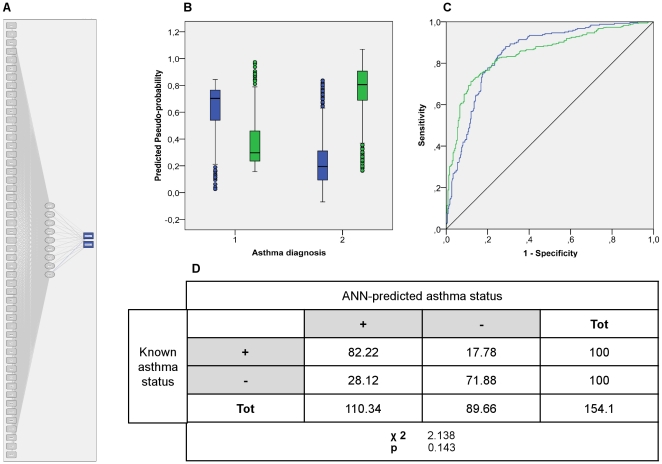
Architecture and performance of the RBF based ANN asthma classifier. (A) ANN architecture. The RBF network consists of three layers: Input (boxes 1–51), hidden (circles 1–8) and output (asthma classes in blue boxes) layer respectively. (B) ANN predicted-by-observed performance chart. The box plots represent the predicted-pseudo-probabilities for the RBF output category; non-asthmatic (green) and asthma (blue) plotted against the known clinical status non-asthmatic (1) asthmatic (2) for combined training and testing samples. (C) The ROC curve calculated on the combined training and testing samples. (D) ANN asthma classifier consistency performance. The ANN-predicted asthma status of the hold out samples was assessed on a Pearson's *X* ^2^ test against the known clinical status of the selected individuals.

The unusually strong association linking some IgE reactivity profiles to asthma prompted us to generate an artificial neural network (ANN) classifier designed to discriminate between asthmatic and non-asthmatic individuals on the basis of the serum reactivity profiles. Each profile used in the supervised training contained information concerning the reaction values for the 51 filtered allergens, and the health status of the individual with respect to the condition of asthma. The ANN correctly classified 82% of the asthmatic patients as “asthmatic” and about 72% of the non-asthmatic as “non-asthmatic” (Figure 3). The overall performance of the ANN was consistent with the results obtained by cluster analysis: the average percentage of asthmatic patients correctly recognized by the RBF classifier as asthmatic is nearly identical to the combined percentage of the asthmatic patients present in clusters 4 and 5. To assess the performance of the ANN-based approach with respect to different classification solutions, we utilized binary logistic regression (BLR). We observed that there was no discrepancy in classification outcome between the two models (data not shown).

**Figure 4:**
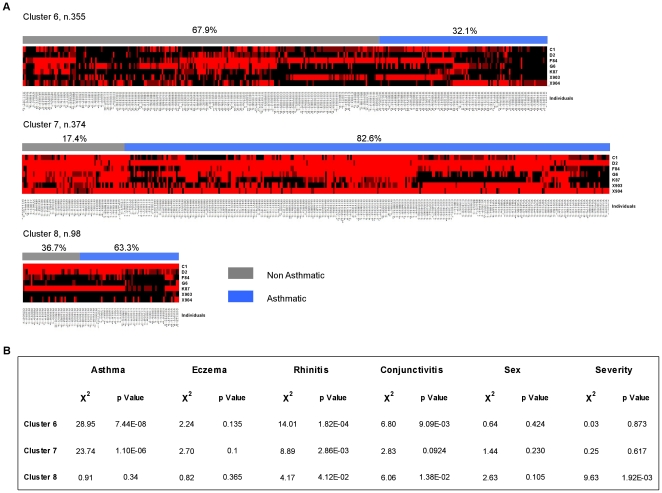
K-means clustering of serum reactivity profiles of 827 sera (columns) against the subset of asthma relevant allergens (rows). (A) The serum reactivity profile generated for each cluster (6–8) at *k* = 3 are visualized. Each column represents an individual serum grouped as asthmatic (blue) and non-asthmatic (gray). Reactivity values to individual allergens are classified as either positive (scale of red - class score 1–5) and negative (black - class score 0). (B) The distribution of atopic conditions in each cluster was assessed using the Pearson's *X* ^2^ test.

Finally, we employed a GEE model [27] to investigate whether the presence of familiar nuclei in the study groups had introduced a bias in the composition of the profiles or had underestimated the standard errors of the analysis. When taking into account the parent to sibling relationships, GEE allows the measurement of population-averaged effects as opposed to cluster-specific effects. We used the GEE univariate analyses to determine association between asthma and IgE reactivity against individual allergens (Table S9). This approach generated a list of 43 relevant allergens with p values <0.05 that coincided substantially with those generated with the Mann-Whitney analysis (forty out of the 43 allergens were identical; 93.02%). To unravel association between distinct reactivity profiles and asthma, a forward multivariate GEE analysis was applied. In this analysis seven allergens (Table 3) were independent predictors of asthma status, while controlling for age, sex and the interaction between age and sex as confounding variables. The final GEE model, factoring in the effect of parent to sibling relationships distinguished asthmatic and non-asthmatic individuals with high accuracy, based on their of the IgE serum reactivity against a reduced number of relevant allergens. The model correctly classified 88.8% of the asthmatic patients as “asthmatic” and 90.9% of the non-asthmatic as “non-asthmatic”. Six out of seven allergens are in common to the 51 relevant allergens generated with the Mann-Whitney procedure. To assess the performance of the GEE-based approach the reactivity profiles against the 7 relevant allergens were utilized to perform clustering association analysis at *k* = 3 (Figure 4). The analysis of the new clusters (6, 7 and 8) showed a striking increase in the statistical significance of the distribution of asthma amongst the new clusters (χ^2^ = 192.549, p = 1.54E-42) (Table S10). The clusters generated with the filtered set of 7 allergens did not show a significant co-segregation of family members with respect to those generated with the all set of allergens (Table S11).

**Table 3:** Results of multivariate GEE analysis.

Variables	Coefficient	S.E.	Wald χ^2^	df	p	OR (95% CI)*
**Penicillin G**					**0.000** †	
C1 - score 4	1.705	0.456	13.967	1	0.000	5.503 (2.25–13.46)
C1 - score 3	−1.143	0.335	11.658	1	0.001	0.319 (0.16–0.61)
C1 - score 2	−0.373	0.175	4.559	1	0.033	0.688 (0.49–0.97)
C1 - score 1	−0.451	0.178	6.444	1	0.011	0.637 (0.45–0.90)
**Derm. Farinae**					**0.000** †	
D2 - score 4	1.460	0.275	28.134	1	0.000	4.305 (2.51–7.38)
D2 - score 3	1.407	0.306	21.088	1	0.000	4.084 (2.24–7.45)
D2 - score 2	0.640	0.200	10.185	1	0.001	1.896 (1.28–2.81)
D2 - score 1	−0.013	0.218	0.004	1	0.951	0.987 (0.64–1.51)
**Kiwi**					**0.000** †	
F84 - score 4	−2.207	0.541	16.619	1	0.000	0.110 (0.04–0.31)
F84 - score 3	0.130	0.299	0.189	1	0.664	1.139 (0.63–2.05)
F84 - score 2	0.484	0.207	5.462	1	0.019	1.623 (1.08–2.43)
F84 - score 1	0.469	0.210	4.985	1	0.026	1.599 (1.06–2.41)
**Timothy grass**					**0.000** †	
G6 - score 4	0.912	0.493	3.413	1	0.065	2.489 (0.95–6.55)
G6 - score 3	0.865	0.310	7.795	1	0.005	2.375 (1.29–4.36)
G6 - score 2	0.604	0.190	10.094	1	0.001	1.829 (1.26–2.65)
G6 - score 1	0.949	0.233	16.628	1	0.000	2.584 (1.63–4.08)
**Alpha amylase**					**0.011** †	
K87 - score 3	−0.808	0.437	3.418	1	0.065	0.446 (0.19–1.05)
K87 - score 2	−0.191	0.226	0.717	1	0.397	0.826 (0.53–1.29)
K87 - score 1	0.458	0.225	4.153	1	0.042	1.581 (1.02–2.46)
**Ph1 p1**					**0.000** †	
X903 - score 4	2.171	0.513	17.880	1	0.000	8.765 (3.20–23.97)
X903 - score 3	0.723	0.244	8.793	1	0.003	2.060 (1.28–3.32)
X903 - score 2	0.328	0.185	3.140	1	0.076	1.388 (0.97–1.99)
X903 - score 1	0.064	0.224	0.081	1	0.776	1.066 (0.69–1.65)
**Derp 1**					**0.038** †	
X904 - score 4	−0.401	0.468	0.733	1	0.392	0.670 (0.27–1.68)
X904 - score 3	0.330	0.253	1.704	1	0.192	1.391 (0.85–2.28)
X904 - score 2	0.208	0.226	0.846	1	0.358	1.231 (0.79–1.92)
X904 - score 1	0.530	0.217	5.984	1	0.003	1.699 (1.11–2.60)

*Odds ratio, confidence intervals and statistical significance were calculated for each class score (4,3,2,1 with respect to class score 0) of specific IgE.

† p values for global effect test.

## Discussion

A complete understanding of the combination of allergens differentially recognized by asthmatic and non-asthmatic individuals would be immensely beneficial for elucidating how specific IgE contribute to the pathogenesis of the disease but progress in this area has been slow because traditional immunoassays such as RAST, CAP ELISA, while useful in assessing specific immune responses, allow for the analysis of just one or few allergens at a time. To overcome these limitations we utilized a microarray immunoassay technology to analyze the IgE reactivity against a vast number of natural and recombinant allergens in the sera of asthmatic and non-asthmatic individuals. This methodology has already been shown to be useful in the serodiagnosis of allergy, infectious diseases, and may soon replace ELISAs in clinical laboratory settings [25], [26], [27], [28]. It has been widely recognized that the analysis of the reactivity profiles not only provides a large amount of quantitative information (the sum of the individual reactivity), but also generates a higher order of knowledge in term of unique combinations of antigen-antibody reactions that associate with different experimental and pathological conditions [26].

Using *k*-means clustering the serum reactivity profiles of all individuals analyzed were arranged in three clusters that, significantly differed from each other in terms of the combination of allergens recognized and in the proportion of individuals affected by different atopic diseases both amongst the case-control groups and the parent-siblings pairs. In particular, asthmatic individuals contributed to 83% of the reactivity profiles of cluster 1. This percentage showed a remarkable statistical significant difference (p<10E-9) compared to that of the asthmatic individuals in the study population. While, cluster 0 and cluster 2 did not show significant differences in the proportion of asthmatic and non-asthmatic individuals, the analysis of their composition demonstrated that, in contrast to cluster 1, they were enriched in members of the same family nuclei. We thought that the composition of cluster 0 and 2 reflected the exposure of family members to a common set of allergens that were included in the microarray that though having a powerful sensitizing ability, were not relevant for asthma and in addition could be due to cross-reactivity [29]. Accordingly, we identified amongst the 103 arrayed allergens a subset of 51 allergens that most differed in the IgE reactivity of asthmatic and non-asthmatic individuals. These allergens were used to generate new reactivity profiles that were clustered using *k*-means. The new clusters showed some interesting and novel features. Cluster 4 was very similar to cluster 1 in terms of IgE recognition pattern and the high proportion of asthmatic individuals. The two remaining clusters also showed highly significant statistical differences in their composition. Cluster 3 was enriched in non-asthmatic individuals (69%) while cluster 5 contained a high percentage of asthmatic patients (82%). The distribution of rhinitis and conjunctivitis (but not eczema) in the clusters closely mirrored that of asthma in agreement with other observations that have established a link amongst these atopic diseases [30], [31]. As anticipated, we did not observe in cluster 3, 4 and 5 any enrichment in members of the same family nuclei thus indicating that the corresponding profiles are associated with the asthma clinical status rather than to allergen exposure. The two clusters that contained the highest proportion of asthmatic individuals (cluster 4 and 5) reacted against the same subset of 21 allergens though cluster 4 showed an additional specific reactivity against nine allergens mainly of food and grasses origin. Notably cluster 4 but not cluster 5 showed an association with asthma severity thus unraveling an unsuspected link between disease severity, on one side and the complexity and the specificity of the IgE response on the other one. This result is consistent with the notion that quantification of IgE antibodies may serve as a marker of severity of asthma [32] and that not only IgE antibodies levels but also the number of allergens reacting positively when tested are needed for the correct classification of disease [33]. GEE statistical analysis showed that the presence of family relationships amongst the individuals enrolled in this study did not account neither for an unequal partitioning nor for an underestimation of standard errors. The GEE multivariate analysis could distinguish asthmatic and non-asthmatic individuals with high accuracy, based on their of the IgE serum reactivity. In this analysis seven allergens were independent predictors of asthma status. Association studies performed on clusters using 7 relevant allergens revealed that the GEE-based approach and the k-means analysis based on the Mann-Whitney selection of allergens gave the same results.

Among the 7 relevant allergens are Dermatophagoides Farinae and Derp1 allergens, this result is not surprising, considering the prevalence rate of both skin prick test (SPT) to Dermatophagoides Pteronissinus and Farinae versus the other SPTs observed among Sardinian people, respectively 82.7% and 76.9% (data not shown).

Timothy grass is a highly prevalent grass worldwide and the prevalence of allergen-specific IgE resulted 8.1–34.6% in the European Community Respiratory Health Survey [34]. Recent studies showed the efficacy of timothy grass allergy immunotherapy tablet treatment in improvement in asthma symptoms both in North American adults and children [35], [36].

The last two relevant allergens included the alpha amylase and the kiwi. The alpha amylase is generally associated with Bakers' asthma, the most common occupational respiratory disease, caused by occupational exposure to the antigens from flour. Interestingly, a potential association between respiratory allergy to cereal flour and allergy to kiwi fruit has been recently disclosed. Cross-reactive carbohydrate determinants and thiol-proteases homologous to Act d1 (cysteine protease) are responsible for wheat-kiwi cross-reactivity in some patients [37].

While this study demonstrates a link between specific IgE and asthma it should be emphasised that a comprehensive understanding of the relationship between asthma and allergen specific IgE would require an exhaustive analysis of reactivity profiles in populations exposed to different set of allergens. The allergens required for such findings would probably vary depending on both subject's age and geographical area. In this context a major advantage of microarray immunoassays is that the composition of different sets of allergens can be expanded and improved continuously, facilitating the identification of the most appropriate reactivity data profile for asthma diagnosis and hence favouring preventive medicine and curative therapies both for asthma and allergic diseases.

Our data demonstrate that associations between asthma and IgE antibody responses to single allergens dramatically underestimate the underlying similarities and differences in individual reactivity to the allergen repertoire that may be relevant for understanding the causes, the severity and the progression of the disease. This also explains why making associations between antibody responses and disease is hard to identify. On the contrary, by analyzing the IgE serum reactivity profiles against a large set of allergens, we could demonstrate that asthmatic and non-asthmatic individuals differ dramatically in terms of number and class of recognised allergens. This information was utilised to train an ANN capable of distinguishing asthmatic and non-asthmatic individuals with high accuracy based on their IgE serum reactivity. This work provides a new framework for understanding the role of allergen specific IgE in the pathogenesis of asthma, thus helping in explaining the occurrence of acute episodes in the apparent absence of exposure to a single allergen, and will prove invaluable in implementing preventive and therapeutic measures.

## Supporting Information

Text S1 **Development of IgE microarray immunoassay.**
(DOC)

Table S1 **List of arrayed allergens grouped according to their source.**
(DOC)

Table S2 **Partition assessment analysis at different** ***k*** **values.**
(DOC)

Table S3 **The case-control group.**
(DOC)

Table S4 **Cluster distribution of case-control reactivity profiles.**
(DOC)

Table S5 **Segregation of family members in the clusters.**
(DOC)

Table S6 **List of asthma relevant allergens.**
(DOC)

Table S7 **Distribution of atopic traits amongst the filtered reactivity profiles.**
(DOC)

Table S8 **Segregation of family nuclei in the filtered clusters.**
(DOC)

Table S9 **Univariate GEE analysis results between asthma and specific IgE against all tested allergens.**
(DOC)

Table S10 **Distribution of atopic traits amongst the reactivity profiles.**
(DOC)

Table S11 **Segregation of family members in the clusters 6–8.**
(DOC)

Figure S1 **Visual representation of the reactivity patterns of cluster 3, 4 and 5.** The numbers on the x axis correspond to the asthma relevant allergens (list on Table S6), the y axis shows the corresponding class score serum reactivity (0–5) in term of mean value red circles), the interquartile range (IQR- blue vertical lines) and the medians (horizontal lines of the IQR).
(DOC)

## References

[1] Fanta CH (2009). Asthma.. N Engl J Med.

[2] Jansson SA, Ronmark E, Forsberg B, Lofgren C, Lindberg A (2007). The economic consequences of asthma among adults in Sweden.. Respir Med.

[3] Seaton A, Godden DJ, Brown K (1994). Increase in asthma: A more toxic environment or a more susceptible population?. Thorax.

[4] Cookson W (1999). The alliance of genes and environment in asthma and allergy.. Nature.

[5] Prescott SL, Macaubas C, Holt BJ, Smallacombe TB, Loh R (1998). Transplacental priming of the human immune system to environmental allergens: Universal skewing of initial t cell responses toward the th2 cytokine profile.. J Immunol.

[6] Stein RT, Sherrill D, Morgan WJ, Holberg CJ, Halonen M (1999). Respiratory syncytial virus in early life and risk of wheeze and allergy by age 13 years.. Lancet.

[7] Halonen M, Stern DA, Lohman C, Wright AL, Brown MA (1999). Two subphenotypes of childhood asthma that differ in maternal and paternal influences on asthma risk.. Am J Respir Crit Care Med.

[8] Venables KM, Chan-Yeung M (1997). Occupational asthma.. Lancet.

[9] Platts-Mills TA (2001). The role of immunoglobulin e in allergy and asthma.. Am J Respir Crit Care Med.

[10] Burrows B, Martinez FD, Halonen M, Barbee RA, Cline MG (1989). Association of asthma with serum IgE levels and skin-test reactivity to allergens.. N Engl J Med.

[11] Menz G, Ying S, Durham SR, Corrigan CJ, Robinson DS (1998). Molecular concepts of IgE -initiated inflammation in atopic and nonatopic asthma.. Allergy.

[12] Platts-Mills TA, Erwin EA, Heymann PW, Woodfolk JA (2009). Pro: The evidence for a causal role of dust mites in asthma.. Am J Respir Crit Care Med.

[13] Platts-Mills TA, Carter MC (1997). Asthma and indoor exposure to allergens.. N Engl J Med.

[14] Tovey ER, Almqvist C, Li Q, Crisafulli D, Marks GB (2008). Nonlinear relationship of mite allergen exposure to mite sensitization and asthma in a birth cohort.. J Allergy Clin Immunol.

[15] Simpson A, Tan VY, Winn J, Svensen M, Bishop CM (2010). Beyond atopy: Multiple patterns of sensitization in relation to asthma in a birth cohort study.. Am J Respir Crit Care Med.

[16] Arshad SH, Tariq SM, Matthews S, Hakim E (2001). Sensitization to common allergens and its association with allergic disorders at age 4 years: A whole population birth cohort study.. Pediatrics.

[17] Mandhane PJ, Sears MR, Poulton R, Greene JM, Lou WY (2009). Cats and dogs and the risk of atopy in childhood and adulthood.. J Allergy Clin Immunol.

[18] Sears MR, Burrows B, Flannery EM, Herbison GP, Holdaway MD (1993). Atopy in childhood. I. Gender and allergen related risks for development of hay fever and asthma.. Clin Exp Allergy.

[19] National Institutes of Health/National Heart, Lung, and Blood Institute (NHLBI), World Health Organization (WHO) (2009). Global initiative for asthma (GINA). Global strategy for asthma management and prevention.

[20] Bacarese-Hamilton T, Mezzasoma L, Ingham C, Ardizzoni A, Rossi R (2002). Detection of allergen-specific IgE on microarrays by use of signal amplification techniques.. Clin Chem.

[21] de Hoon MJ, Imoto S, Nolan J, Miyano S (2004). Open source clustering software.. Bioinformatics.

[22] Simirenko L (2004).

[23] Bolshakova N, Azuaje F, Cunningham P (2005). An integrated tool for microarray data clustering and cluster validity assessment.. Bioinformatics.

[24] Bishop CM (1995). Neural networks for pattern recognition.

[25] Zeger SL, Liang KY (1986). Longitudinal data analysis for discrete and continuous outcomes.. Biometrics.

[26] Gray JC, Corran PH, Mangia E, Gaunt MW, Li Q (2007). Profiling the antibody immune response against blood stage malaria vaccine candidates.. Clin Chem.

[27] Bacarese-Hamilton T, Mezzasoma L, Ardizzoni A, Bistoni F, Crisanti A (2004). Serodiagnosis of infectious diseases with antigen microarrays.. J Appl Microbiol.

[28] Mullenix MC, Whiltshire S, Shao W, Kitos G, Schweitzer B (2001). Allergen-specific IgE detection on microarrays using rolling circle amplification: Correlation with in vitro assays for serum IgE.. Clinical Chemistry.

[29] Alberse RC, Akkerdaas J, van Ree R (2001). Cross-reactivity of IgE antibodies to allergens.. Allergy.

[30] The international study of asthma and allergies in childhood (ISAAC) steering committee (1998). Worldwide variation in prevalence of symptoms of asthma, allergic rhinoconjunctivitis, and atopic eczema: ISAAC.. Lancet.

[31] Ker J, Hartert TV (2009). The atopic march: What's the evidence?. Ann Allergy Asthma Immunol.

[32] Wickman M (2004). Experience with quantitative IgE antibody analysis in relation to allergic disease within the BAMSE birth cohort-towards an improved diagnostic process.. Allergy.

[33] Wickman M, Lilja G, Soderstrom L, van HageHamsten M, Ahlsted S (2005). Quantitative analysis of IgE antibodies to food and inhalant in 4-year-old children reflects their likelihood of allergic disease.. Allergy.

[34] Burney B, Malmberg E, Chinn S, Jarvis D, Luczynska C (1997). The distribution of total and specific serum IgE in the European Community Respiratory Health Survey.. J Allergy Clin Immunol.

[35] Nelson A, Nolte H, Creticos P, Maloney J, Wu J (2011). J Allergy Clin Immunol.

[36] Blaiss M, Maloney J, Nolte H, Gawchik G, Yao R (2011). J Allergy Clin Immunol.

[37] Palacin A, Quirce S, Sanchez-Monge R, Fernandez-Nieto M, Varala J (2008). Allergy to kiwi in patients with baker's asthma: identification of potential cross-reactive allergens.. Ann Allergy Asthma Immunol.

