# Supplementary material for: Serum IgE Reactivity Profiling in an Asthma Affected Cohort
Source: PLoS One. 2011 Aug 4;6(8):e22319. doi: 10.1371/journal.pone.0022319 (PMC3150333; doi:10.1371/journal.pone.0022319)
Supplement: Text S1 — Development of IgE microarray immunoassay. (DOC) [file pone.0022319.s001.doc]

**Text S1. Development of IgE microarray immunoassay.**

To generate the array the selected allergens (natural extracts, purified allergens and recombinant molecules) were printed onto aldehyde-activated glass microscope slides in duplicates at scrambled positions to minimize the effect of processing errors. The microarray immunoassay procedure consisted of four phases (*printing*, *processing, scanning, quantification and analysis).*

Printing of microarrays: To reduce the standard spotting time, decrease the inter-batch variability and increase the number of chip processed per test, 2 chips (“allergochip”) were printed onto single microscope slide. Each microarray batch included 120 slides, for a total of 240 allergochips (execution time is 14 hours). The analysis of the study group required a total of 480 printed slides, corresponding to 960 chips, the overall printing procedure was divided in 4 batches. Each chip contained 103 allergens printed in duplicate onto aldehyde-activated glass microscope slides (CEL Associates) using high –speed robotics (Microgrid Compact; Biorobotics). Arrays were printed at 23**°**C and 60% humidity and stored overnight inside the printing cabinet. Allergens (provided from Allergopharma) were initially reconstituted in PBS pH7.4 (reconstitution buffer) with a final concentration ranging form 0.4 to 40 mg/ml and after that spotted onto the arrays in the following spotting buffer: PBS pH7.4, glycine pH2.4, Borate pH9.4, glycerol 10%, DTT 5mM, SDS (0.2%; 0.05%), Tween 20 (0.01%). Allergens spotting concentration ranged from 0.008 to 3 mg/ml. A detailed description of the sample (allergens, stock and spotting concentration, spotting buffer) is provided upon request.

Assay protocol: Printed slides were blocked with PBS containing 2% BSA for 1 h at room temperature. Slides were then incubated with serum samples (100 µl) for 60 minutes at 37**°**C. To reveal bound IgE, the slides were incubated with a secondary mouse monoclonal antibody directed against human IgE (0.14 µg/ml - 100µl) for 45 minutes at 37**°**C, followed by an incubation with anti-mouse IgG HRP conjugated antibody (1.6 µg/mL - 100µl) for 45 minutes at 37**°**C and finally incubated with tyramide-Alexa 555 (TSA™ Kit #42 *with HRP—streptavidin and Alexa Fluor® 555 tyramide* *50-150 slides*) (Invitrogen) diluted 1:200 (100µl), for 15 minutes at 37**°**C. Slides were dried at 37**°**C before measuring the fluorescence signal.

Reading assay results: The processed slides were scanned using a fluorescence-detecting scanner ScanArray™ Gx and the images were generated with the ScanArray™ software provided by Perkin Elmer Life Sciences Inc. All the slides were scanned under identical settings: 90 % laser power and 60 % photomultiplier gain.

Quantification of bound IgE: The fluorescence signal was acquired using ProScanArray Express™ version 3.0 software. PMC reading values of individual spots were corrected against the internal negative control to identify signals above background. Duplicate measurements of individual allergens were utilized. Concentrations (IU/ml) of allergen- bound IgE were determined by interpolation with an internal calibration curve printed onto each microarray. The calibration curve consisted of decreasing amounts of streptavidin (80; 53.3; 35.6; 23.7; 15.8; 10.5; 7.02 µg/ml) that capture myeloma biotinylated IgE spiked into the blocking solution. To assign IU/ml values to the calibration curve, the average signals collected at different amounts of printed streptavidin were interpolated with an external Reference Curve generated by microarray slides printed with replicates of Goat anti-Human IgE and incubated with increasing concentrations of human IgE (WHO Reference standard 0.35, 1.0, 3.5, 10.0, 50.0 IU/ml). The signal collected from the allergens was interpolated with the calibration curve to obtain the IU/ml value, and translated into a Class Score by plotting the data in a standard reactivity scale. Class Score values: (CLASS 0 (less than 0.35 IU/ml); CLASS 1 (0.35-0.7 IU/ml); CLASS 2 (0.71-3.5 IU/ml); CLASS 3 (3.51-17.5 IU/ml); CLASS 4 (17.51-50 IU/ml); CLASS 5 (50.01-100 IU/ml).
