# Supplementary material for: Serum IgE Reactivity Profiling in an Asthma Affected Cohort
Source: PLoS One. 2011 Aug 4;6(8):e22319. doi: 10.1371/journal.pone.0022319 (PMC3150333; doi:10.1371/journal.pone.0022319)
Supplement: Table S1 — List of arrayed allergens grouped according to their source. (DOC) [file pone.0022319.s002.doc]

**Table S1.** List of arrayed allergens grouped according to their source.

| **Drugs** | F44 (Strawberry) | M2 (*Cladorporium erbarum*) |
| --- | --- | --- |
| C1 (Penicillin G) | F45 (Baker’s yeast) | M3 (*Aspergillus fumigatus*) |
| C2 (Penicillin V) | F46 (Pepper) | M4 (*Mucor racemosus*) |
| C214 (Amoxicillin | F49 (Apple) | M5 (*Candida albicans)* |
| **Mites** | F52 (Chocolate) | M6 (*Alternaria tenuis*) |
| D1 (*Dermatophagoides pteronyssinus*) | F74 (Hen’s egg) | M7 (*Botrytis cinerea*) |
| D2 (*Dermatophagoides farinae*) | F76 (Alpha-Lactalbumin) | M9 (*Fusarium moniliforme*) |
| D3 (*Dermatophagoides microceras*) | F77 (β-Lactoglobulin) | M13 (*Phoma betae*) |
| D70 (*Acarus siro*) | F78 (Casein) | M20 (*Mucor mucedo*) |
| D71 (*Lepidoglyfus destructor*) | F83 (Chicken meat) | **Tree pollens** |
| D72 (*Tyrophagus putrescentia*e) | F84 (Kiwi) | T2 (Alder) |
| D73 (*Glyciphagus domesticus*) | F85 (Celery) | T3 (Birch pollen) |
| **Animal epithelia** | F92 (Banana) | T4 (Hazel) |
| E1 (Cat hair) | F95 (Peach) | T5 (European beech) |
| E2 (Dog hair) | **Grass pollens** | T6 (Mountain cedar) |
| E3 (Horse hair) | G1 (Sweet vernal grass) | T7 (Oak) |
| E6 (Guinea pig epithelium) | G2 (Bermuda grass/squitch) | T9 (Olive) |
| E78 (Budgerigar feathers) | G3 (Orchard grass) | T11 (Plane) |
| E81 (Sheep epithelium) | G4 (Meadow fescue) | T14 (Poplar) |
| E82 (Rabbit epithelium) | G5 (Ryegrass perennial) | T901 (Ash) |
| **Food allergens** | G6 (Timothy grass) | T904 (Sallow) |
| F1 (Egg white) | G8 (Bluegrass, June – Kentucky) | **Weed pollens** |
| F2 (Cow’s milk) | G12 (Rye cultivated) | W1 (Ragweed common) |
| F3 (Cod) | G14 (Oats cultivated) | W6 (Mugwort) |
| F4 (Wheat flour) | G15 (Wheat) | W8 (Dandelion) |
| F7 (Oat flour) | G18 (Barley) | W9 (English plantain) |
| F8 (Corn flour) | **Insects** | W20 (Stinging nettle) |
| F13 (Peanuts) | I1 (Honeybee venom) | W21 (Parietaria) |
| F14 (Soybean) | I3 (Wasp venom) | W32 (Rape) |
| F16 (Walnut) | I6 (Cockroach) | **Purified proteins** |
| F17 (Hazelnut) | I71 (Midge/Mosquito/Gnat) | X901 Bet v 1 |
| F23 (Shrimp) | **Occupational allergens** | X902 Phl p 5 (G6-V) |
| F25 (Tomato) | K81 (Ficus benjamina) | X903 Phl p 1 |
| F26 (Pork) | K82 (Latex) | X904 Der p 1 (D1-I) |
| F27 (Beef) | K87 (Alpha amylase) | X905 Der p 2 (D1-II) |
| F31 (Carrot) | K905 (HSA) | X907 Bet v 2 |
| F33 (Orange) | **Moulds** | X910 Phl p 2 |
| F35 (Potato) | M1 (*Penicillium notatum*) | X911 Phl p 6 |
