# Supplementary material for: Serum IgE Reactivity Profiling in an Asthma Affected Cohort
Source: PLoS One. 2011 Aug 4;6(8):e22319. doi: 10.1371/journal.pone.0022319 (PMC3150333; doi:10.1371/journal.pone.0022319)
Supplement: Table S2 — Partition assessment analysis at different k values. (DOC) [file pone.0022319.s003.doc]

**Table S2**. **Partition assessment analysis at different *k* values**.

| **K value** | k=3 | k=4 | k=5 | k=6 | k=7 | k=8 | k=9 | k=10 | k=11 | k=12 | k=13 | k=14 | k=20 |
| --- | --- | --- | --- | --- | --- | --- | --- | --- | --- | --- | --- | --- | --- |
| **Validity index*** |  |  |  |  |  |  |  |  |  |  |  |  |  |
| **Silhouette** | 0.183^‡^ | 0.164 | 0.173 | 0.167 | 0.173 | 0.168 | 0.146 | 0.150 | 0.154 | 0.154 | 0.152 | 0.151 | 0.154 |
| **Dunn** | 0.846^‡^ | 0.833 | 0.807 | 0.696 | 0.708 | 0.651 | 0.648 | 0.628 | 0.628 | 0.670 | 0.622 | 0.622 | 0.666 |
| **Davies Bouldin** | 1.730^‡^ | 1.756 | 1.733 | 1.812 | 1.769 | 1.789 | 1.797 | 1.783 | 1.804 | 1.780 | 1.781 | 1.735 | 1.732 |
| **C** | 0.138 | 0.162 | 0.142 | 0.145 | 0.122 | 0.117 | 0.127 | 0.127 | 0.114 | 0.102 | 0.103 | 0.098 | 0.076 |
| **Isolation** | 0.837^‡^ | 0.796 | 0.779 | 0.748 | 0.717 | 0.685 | 0.661 | 0.658 | 0.647 | 0.627 | 0.635 | 0.626 | 0.581 |

*Validity indexes assess different cluster parameters such as tightness, separation and compactness (see methods). Euclidean metric was applied to the analysis of all parameters

^‡^ Best partition results
