# Supplementary material for: Serum IgE Reactivity Profiling in an Asthma Affected Cohort
Source: PLoS One. 2011 Aug 4;6(8):e22319. doi: 10.1371/journal.pone.0022319 (PMC3150333; doi:10.1371/journal.pone.0022319)
Supplement: Table S3 — The case-control group. (DOC) [file pone.0022319.s004.doc]

**Table S3. The case-control group.**

| **Asthmatic Adults** | **Sex^*^** | **Age** | **Non-asthmatic Adults** | **Sex** | **Age** | **∆** |
| --- | --- | --- | --- | --- | --- | --- |
| 1 | M | 31 | 1 | M | 31 | 0 |
| 2 | M | 35 | 2 | M | 35 | 0 |
| 3 | M | 37 | 3 | M | 37 | 0 |
| 4 | M | 37.9 | 4 | M | 38 | -0.1 |
| 5 | M | 41 | 5 | M | 41 | 0 |
| 6 | M | 41 | 6 | M | 41 | 0 |
| 7 | M | 42 | 7 | M | 42 | 0 |
| 8 | M | 42.9 | 8 | M | 42.9 | 0 |
| 9 | M | 43 | 9 | M | 43 | 0 |
| 10 | M | 43 | 10 | M | 43 | 0 |
| 11 | M | 43 | 11 | M | 43.1 | -0.1 |
| 12 | M | 43.5 | 12 | M | 43.9 | -0.4 |
| 13 | M | 45 | 13 | M | 45 | 0 |
| 14 | M | 48 | 14 | M | 48 | 0 |
| 15 | M | 50 | 15 | M | 50 | 0 |
| 16 | M | 51 | 16 | M | 51 | 0 |
| 17 | M | 52 | 17 | M | 52 | 0 |
| 18 | M | 54 | 18 | M | 54 | 0 |
| 19 | M | 54 | 19 | M | 54 | 0 |
| 20 | M | 55 | 20 | M | 55 | 0 |
| 21 | M | 55.4 | 21 | M | 55.2 | 0.2 |
| 22 | M | 56 | 22 | M | 56 | 0 |
| 23 | M | 56 | 23 | M | 56 | 0 |
| 24 | M | 60.8 | 24 | M | 60.7 | 0.1 |
| 25 | M | 62.3 | 25 | M | 62.2 | 0.1 |
| 26 | M | 63 | 26 | M | 63 | 0 |
| 27 | F | 31 | 27 | F | 31 | 0 |
| 28 | F | 31 | 28 | F | 31 | 0 |
| 29 | F | 32 | 29 | F | 32 | 0 |
| 30 | F | 32.3 | 30 | F | 32 | 0.3 |
| 31 | F | 36.1 | 31 | F | 36.2 | -0.1 |
| 32 | F | 36.6 | 32 | F | 36.2 | 0.4 |
| 33 | F | 37 | 33 | F | 37 | 0 |
| 34 | F | 37.3 | 34 | F | 37.3 | 0 |
| 35 | F | 39 | 35 | F | 39 | 0 |
| 36 | F | 40 | 36 | F | 40 | 0 |
| 37 | F | 40 | 37 | F | 40 | 0 |
| 38 | F | 41 | 38 | F | 41 | 0 |
| 39 | F | 41.9 | 39 | F | 41.5 | 0.4 |
| 40 | F | 42 | 40 | F | 42 | 0 |
| 41 | F | 45 | 41 | F | 45 | 0 |
| 42 | F | 47 | 42 | F | 47 | 0 |
| 43 | F | 48.1 | 43 | F | 48.1 | 0 |
| 44 | F | 49.9 | 44 | F | 50 | -0.1 |
| 45 | F | 52.1 | 45 | F | 52 | 0.1 |
| 46 | F | 53 | 46 | F | 53 | 0 |
| 47 | F | 54.2 | 47 | F | 54.2 | 0 |
| 48 | F | 59 | 48 | F | 58.3 | 0.7 |
| 49 | F | 59.2 | 49 | F | 60.3 | -1.1 |
| 50 | F | 60 | 50 | F | 60.3 | -0.3 |
| 51 | F | 60.8 | 51 | F | 60.7 | 0.1 |
| 52 | F | 61 | 52 | F | 61 | 0 |
| 53 | F | 61.4 | 53 | F | 61.4 | 0 |
| 54 | F | 61.6 | 54 | F | 64 | -2.4 |
| 55 | F | 67 | 55 | F | 67 | 0 |
| 56 | F | 71 | 56 | F | 72 | -1 |
| 57 | F | 73 | 57 | F | 72 | 1 |
|  |  |  |  |  | **∆ age mean** | **-0.038596491** |
|  |  |  |  |  | **∆ age std.dev** | **0.425841346** |

*M=Male, F=Female
