# Supplementary material for: Serum IgE Reactivity Profiling in an Asthma Affected Cohort
Source: PLoS One. 2011 Aug 4;6(8):e22319. doi: 10.1371/journal.pone.0022319 (PMC3150333; doi:10.1371/journal.pone.0022319)
Supplement: Table S4 — Cluster distribution of case-control reactivity profiles. (DOC) [file pone.0022319.s005.doc]

**Table S4. Cluster distribution of case-control reactivity profiles**

|  | **Asthma** | |
| --- | --- | --- |
| **Allergens = 103^*^** | - | + |
| Cluster 0 | 69.6% | 30.4% |
| Cluster 1 | 18.8% | 81.3% |
| Cluster 2 | 50.7% | 49.3% |
| **Total** | 50.0% | 50.0% |
| **χ2** | 9.785 | |
| **p-value** | 7.50E-03 | |

^*^Number of allergens utilized to generate the profiles of clusters 0-2
