# Supplementary material for: Serum IgE Reactivity Profiling in an Asthma Affected Cohort
Source: PLoS One. 2011 Aug 4;6(8):e22319. doi: 10.1371/journal.pone.0022319 (PMC3150333; doi:10.1371/journal.pone.0022319)
Supplement: Table S5 — Segregation of family members in the clusters. (DOC) [file pone.0022319.s006.doc]

Table S5. Segregation of family members in the clusters.

| **allergens n= 103**^*^ | **F** | **FC** | **FCC** | **FCCC** | **FM** | **FMC** | **FMCC** | **FMCCC** | **M** | **MC** | **MCC** | **C** | **CC** | **CCC** |
| --- | --- | --- | --- | --- | --- | --- | --- | --- | --- | --- | --- | --- | --- | --- |
| **Cluster 0** | 3 | 5 | 1 | 0 | 3 | 18 | 20 | 0 | 2 | 7 | 5 | 5 | 1 | 0 |
| **Cluster 1** | 13 | 4 | 2 | 0 | 0 | 0 | 0 | 0 | 6 | 11 | 2 | 60 | 12 | 1 |
| **Cluster 2** | 4 | 6 | 3 | 0 | 24 | 64 | 26 | 3 | 13 | 24 | 13 | 33 | 7 | 0 |

^*^Number of allergens utilized to generate the profiles of clusters 0-2

F=father, M=mother, C=children, FC= father and child, FCC=father and 2 children , FCCC=father and 3 children, FM=father and mother, FMC=father, mother and children, FMCC=father, mother and 2 children, FMCCC=father, mother and 3 children, MC= mother and children, MCC=mother and 2 children, CC= 2 children and CCC= 3 children
