# Supplementary material for: Serum IgE Reactivity Profiling in an Asthma Affected Cohort
Source: PLoS One. 2011 Aug 4;6(8):e22319. doi: 10.1371/journal.pone.0022319 (PMC3150333; doi:10.1371/journal.pone.0022319)
Supplement: Table S6 — List of asthma relevant allergens. (DOC) [file pone.0022319.s007.doc]

**Table S6**. **List of asthma relevant allergens**.

| **Drugs** | 18. F84 (Kiwi) | 35. M4 (*Mucor racemosus*) |
| --- | --- | --- |
| 1. C2 (Penicillin V) | 19. F95 (Peach) | 36. M5 (*Candida albicans)* |
| **Mites** | **Grass pollens** | 37. M6 (*Alternaria tenuis*) |
| 2. D1 (*Dermatophagoides pteronyssinus*) | 20. G1 (Sweet vernal grass) | **Tree pollens** |
| 3. D2 (*Dermatophagoides farinae*) | 21. G2 (Bermuda grass/squitch) | 38. T4 (Hazel) |
| 4. D3 (*Dermatophagoides microceras*) | 22. G3 (Orchard grass) | 39. T6 (Mountain cedar) |
| 5. D70 (*Acarus siro*) | 23. G4 (Meadow fescue) | 40. T7 (Oak) |
| 6. D71 (*Lepidoglyfus destructor*) | 24. G5 (Ryegrass perennial) | 41. T9 (Olive) |
| 7. D72 (*Tyrophagus putrescentia*e) | 25. G6 (Timothy grass) | 42. T14 (Poplar) |
| 8. D73 (*Glyciphagus domesticus*) | 26. G8 (Blue grass, June – Kentucky) | 43. T901 (Ash) |
| **Animal Epithelia** | 27. G12 (Rye cultivated) | **Weed pollens** |
| 9. E1 (Cat hair) | 28. G14 (Oats cultivated) | 44. W1 (Ragweed common) |
| 10. E3 (Horse hair) | 29. G15 (Wheat) | 45. W6 (Mugwort) |
| 11. E81 (Sheep epithelium) | 30. G18 (Barley) | **Purified proteins** |
| 12 E82 (Rabbit epithelium) | **Insects** | 46. X902 Phl p 5 (G6-V) |
| **Food allergens** | 31. I6 (Cockroach) | 47. X903 Phl p 1 |
| 13. F4 (Wheat flour) | **Occupational allergens** | 48. X904 Der p 1 (D1-I) |
| 14. F16 (Walnut) | 32. K87 (Alpha amylase) | 49. X905 Der p 2 (D1-II) |
| 15. F25 (Tomato) | **Moulds** | 50. X907 Bet v 2 |
| 16. F35 (Potato) | 33. M1 (*Penicillium notatum*) | 51. X910 Phl p 2 |
| 17. F49 (Apple) | 34. M3 (*Aspergillus fumigatus*) |  |
