# Supplementary material for: Serum IgE Reactivity Profiling in an Asthma Affected Cohort
Source: PLoS One. 2011 Aug 4;6(8):e22319. doi: 10.1371/journal.pone.0022319 (PMC3150333; doi:10.1371/journal.pone.0022319)
Supplement: Table S7 — Distribution of atopic traits amongst the filtered reactivity profiles. (DOC) [file pone.0022319.s008.doc]

**Table S7. Distribution of atopic traits amongst the filtered reactivity profiles.**

|  | **Asthma** | | **Conjunctivitis** | | **Eczema** | | **Rhinitis** | | **Sex** | | **Persistency** | | | **Severity**† | | | **Age onset^‡^** |
| --- | --- | --- | --- | --- | --- | --- | --- | --- | --- | --- | --- | --- | --- | --- | --- | --- | --- |
| **allergens**  **= 51*** | - | + | - | + | - | + | - | + | M | F | - | + | 1 | | 2 |  | |
| **Cluster 3** | 68.9% | 31.1% | 78.8% | 21.2% | 87.6% | 12.4% | 66.8% | 33.2% | 44.3% | 55.7% | 11.4% | 88.6% | 53.7% | | 46.3% | 9.00 | |
| **Cluster 4** | 12.2% | 87.8% | 37.7% | 62.3% | 65.5% | 34.5% | 28.9% | 71.1% | 65.0% | 35.0% | 13.2% | 86.8% | 36.3% | | 63.7% | 7.00 | |
| **Cluster 5** | 17.8% | 82.2% | 60.4% | 39.6% | 73.3% | 26.7% | 32.6% | 67.4% | 54.0% | 46.0% | 9.8% | 90.2% | 50.4% | | 49.6% | 6.00 | |
| **Total** | 41.4% | 58.6% | 65.5% | 34.5% | 78.9% | 21.1% | 48.2% | 51.8% | 51.0% | 49.0% | 10.9% | 89.1% | 47.9% | | 52.1% |  | |
| **χ2** | 237.041 | | 69.377 | | 34.941 | | 99.712 | | 17.937 | | 0.655 | | | 6.616 | | | 6.689 |
| **p-value** | 3.37E-52 | | 8.61E-16 | | 2.59E-08 | | 2.23E-22 | | 1.27E-04 | | 0.721 | | | 0.037 | | | 0.035 |

^*^Number of allergens utilized to generate the profiles of cluster 3-5

† Asthma severity was classified by a physician in four levels according to the World Health Organization guidelines (Global Initiative for Asthma). For simplicity, to better highlight strait differences we considered individuals being of level 1 to 2 as one group (column 1) and individuals with higher severity, level from 3 to 4, as one group (column 2).

^‡^Median values
