# Supplementary material for: Serum IgE Reactivity Profiling in an Asthma Affected Cohort
Source: PLoS One. 2011 Aug 4;6(8):e22319. doi: 10.1371/journal.pone.0022319 (PMC3150333; doi:10.1371/journal.pone.0022319)
Supplement: Table S9 — Univariate GEE analysis results between asthma and specific IgE against all tested allergens. (DOC) [file pone.0022319.s010.doc]

**Table S9. Univariate GEE analysis results between asthma and specific IgE against all tested allergens.**

| Allergens | p value* |
| --- | --- |
| Drugs |  |
| C1 (Penicillin G) | **0.006** |
| C2 (Penicillin V) | 0.621 |
| C214 (Amoxicillin) | 0.881 |
| Mites |  |
| D1 (Dermatophagoides pteronyssinus) | **< 10^-3^** |
| D2 (Dermatophagoides farinae) | **< 10^-3^** |
| D3 (Dermatophagoides microceras) | **< 10^-3^** |
| D70 (Acarus siro) | **0.001** |
| D71 (Lepidoglyfus destructor) | **0.005** |
| D72 (Tyrophagus putrescentiae) | **< 10^-3^** |
| D73 (Glyciphagus domesticus) | **< 10^-3^** |
| Animal epithelia |  |
| E1 (Cat hair) | **< 10^-3^** |
| E2 (Dog hair) | 0.133 |
| E3 (Horse hair) | 0.081 |
| E6 (Guinea pig epithelium) | 0.205 |
| E78 (Budgerigar feathers) | 0.482 |
| E81 (Sheep epithelium) | **0.007** |
| E82 (Rabbit epithelium) | 0.131 |
| Food allergens |  |
| F1 (Egg white) | **0.035** |
| F2 (Cow’s milk) | 0.584 |
| F3 (Cod) | 0.717 |
| F4 (Wheat flour) | 0.099 |
| F7 (Oat flour) | 0.072 |
| F8 (Corn flour) | 0.956 |
| F13 (Peanuts) | 0.379 |
| F14 (Soybean) | 0.264 |
| F16 (Walnut) | **0.019** |
| F17 (Hazelnut) | 0.439 |
| F23 (Shrimp) | 0.344 |
| F25 (Tomato) | 0.067 |
| F26 (Pork) | 0.054 |
| F27 (Beef) | 0.781 |
| F31 (Carrot) | 0.174 |
| F33 (Orange) | 0.659 |
| F35 (Potato) | **0.025** |
| F44 (Strawberry) | 0.942 |
| F45 (Baker’s yeast) | 0.814 |
| F46 (Pepper) | 0.605 |
| F49 (Apple) | **< 10^-3^** |
| F52 (Chocolate) | 0.496 |
| F74 (Hen’s egg) | 0.937 |
| F76 (Alpha-Lactalbumin) | 0.905 |
| F77 (β-Lactoglobulin) | 0.461 |
| F78 (Casein) | 0.235 |
| F83 (Chicken meat) | 0.331 |
| F84 (Kiwi) | **0.016** |
| F85 (Celery) | 0.206 |
| F92 (Banana) | 0.930 |
| F95 (Peach) | **< 10^-3^** |
| Grass pollens |  |
| G1 (Sweet vernal grass) | **< 10^-3^** |
| G2 (Bermuda grass/squitch) | **< 10^-3^** |
| G3 (Orchard grass) | **< 10^-3^** |
| G4 (Meadow fescue) | **< 10^-3^** |
| G5 (Ryegrass perennial) | **< 10^-3^** |
| G6 (Timothy grass) | **< 10^-3^** |
| G8 (Bluegrass, June – Kentucky) | **< 10^-3^** |
| G12 (Rye cultivated) | **< 10^-3^** |
| G14 (Oats cultivated) | **< 10^-3^** |
| G15 (Wheat) | **< 10^-3^** |
| G18 (Barley) | **< 10^-3^** |
| Insects |  |
| I1 (Honeybee venom) | 0.520 |
| I3 (Wasp venom) | 0.482 |
| I6 (Cockroach) | 0.162 |
| I71 (Midge/Mosquito/Gnat) | 0.097 |
| Occupational allergens |  |
| K81 (Ficus benjamina) | 0.303 |
| K82 (Latex) | 0.847 |
| K87 (Alpha amylase) | **0.026** |
| K905 (HSA) | 0.056 |
| Moulds |  |
| M1 (Penicillium notatum) | 0.053 |
| M2 (Cladorporium erbarum) | 0.354 |
| M3 (Aspergillus fumigatus) | **0.017** |
| M4 (Mucor racemosus) | **< 10^-3^** |
| M5 (Candida albicans) | **0.040** |
| M6 (Alternaria tenuis) | **< 10^-3^** |
| M7 (Botrytis cinerea) | 0.591 |
| M9 (Fusarium moniliforme) | 0.640 |
| M13 (Phoma betae) | 0.237 |
| M20 (Mucor mucedo) | 0.659 |
| Tree pollens |  |
| T2 (Alder) | 0.504 |
| T3 (Birch pollen) | 0.760 |
| T4 (Hazel) | 0.079 |
| T5 (European beech) | 0.053 |
| T6 (Mountain cedar) | **0.020** |
| T7 (Oak) | **0.001** |
| T9 (Olive) | **< 10^-3^** |
| T11 (Plane) | 0.515 |
| T14 (Poplar) | **0.004** |
| T901 (Ash) | **0.001** |
| T904 (Sallow) | 0.213 |
| Weed pollens |  |
| W1 (Ragweed common) | 0.084 |
| W6 (Mugwort) | 0.180 |
| W8 (Dandelion) | 0.267 |
| W9 (English plantain) | 0.091 |
| W20 (Stinging nettle) | 0.341 |
| W21 (Parietaria) | 0.098 |
| W32 (Rape) | 0.904 |
| Purified proteins |  |
| X901 Bet v 1 | 0.832 |
| X902 Phl p 5 (G6-V) | **< 10^-3^** |
| X903 Phl p 1 | **< 10^-3^** |
| X904 Der p 1 (D1-I) | **< 10^-3^** |
| X905 Der p 2 (D1-II) | **< 10^-3^** |
| X907 Bet v 2 | **0.004** |
| X910 Phl p 2 | **< 10^-3^** |
| X911 Phl p 6 | 0.126 |

*p values < 0.05 are shown in bold
