# Supplementary material for: Serum IgE Reactivity Profiling in an Asthma Affected Cohort
Source: PLoS One. 2011 Aug 4;6(8):e22319. doi: 10.1371/journal.pone.0022319 (PMC3150333; doi:10.1371/journal.pone.0022319)
Supplement: Table S10 — Distribution of atopic traits amongst the reactivity profiles. (DOC) [file pone.0022319.s011.doc]

**Table S10.** Distribution of atopic traits amongst the reactivity profiles.

|  | **Asthma** | | **Conjunctivitis** | | **Eczema** | | **Rhinitis** | | **Sex** | | **Severity^†^** | |
| --- | --- | --- | --- | --- | --- | --- | --- | --- | --- | --- | --- | --- |
| **allergens**  **= 7*** | - | + | - | + | - | + | - | + | M | F | 1 | 2 |
| **Cluster 6** | 67.9% | 32.1% | 77.9% | 22.1% | 85.0% | 15.0% | 66.9% | 33.1% | 47.0% | 53.0% | 48.7% | 51.3% |
| **Cluster 7** | 17.4% | 82.6% | 57.5% | 42.5% | 72.2% | 27.8% | 33.3% | 66.7% | 57.0% | 43.0% | 45.4% | 54.6% |
| **Cluster 8** | 36.7% | 63.3% | 53.8% | 46.2% | 82.6% | 17.4% | 38.0% | 62.0% | 42.9% | 57.1% | 63.4% | 36.6% |
| **Total** | 41.4% | 58.6% | 65.5% | 34.5% | 78.9% | 21.1% | 48.2% | 51.8% | 51.0% | 49.0% | 47.9% | 52.1% |
| **χ2** | 192.549 | | 35.899 | | 17.78 | | 83.496 | | 10.127 | | 4.687 | |
| **p-value** | 1.54E-42 | | 1.60E-08 | | 1.38E-04 | | 7.40E-19 | | 6.32E-03 | | 9.60E-02 | |

**^*^**Number of allergens utilized to generate the profiles of clusters 6-8

**^†^**Asthma severity was classified by a physician in four levels according to the World Health Organization guidelines (Global Initiative for Asthma). For simplicity, to better highlight strait differences we considered individuals being of level 1 to 2 as one group (column 1) and individuals with higher severity, level from 3 to 4, as one group (column 2).
