# Supplementary material for: Serum IgE Reactivity Profiling in an Asthma Affected Cohort
Source: PLoS One. 2011 Aug 4;6(8):e22319. doi: 10.1371/journal.pone.0022319 (PMC3150333; doi:10.1371/journal.pone.0022319)
Supplement: Table S11 — Segregation of family members in the clusters 6–8. (DOC) [file pone.0022319.s012.doc]

Table S11. Segregation of family members in the clusters 6-8.

| **allergens n= 7*** | **F** | **FC** | **FCC** | **FCCC** | **FM** | **FMC** | **FMCC** | **FMCCC** | **M** | **MC** | **MCC** | **MCCC** | **C** | **CC** | **CCC** | **CCCC** |
| --- | --- | --- | --- | --- | --- | --- | --- | --- | --- | --- | --- | --- | --- | --- | --- | --- |
| **Cluster 6** | 20 | 3 | 5 | 0 | 71 | 21 | 4 | 0 | 35 | 15 | 2 | 0 | 14 | 4 | 0 | 0 |
| **Cluster 7** | 6 | 8 | 3 | 3 | 2 | 5 | 10 | 1 | 5 | 10 | 10 | 1 | 118 | 40 | 1 | 1 |
| **Cluster 8** | 3 | 2 | 1 | 0 | 1 | 4 | 8 | 0 | 1 | 4 | 4 | 0 | 11 | 2 | 0 | 0 |

*Number of allergens utilized to generate the profiles of clusters 6-8

F=father, M=mother, C=children, FC= father and child, FCC=father and 2 children , FCCC=father and 3 children, FM=father and mother, FMC=father, mother and children, FMCC=father, mother and 2 children, FMCCC=father, mother and 3 children, MC= mother and children, MCC=mother and 2 children, CC= 2 children and CCC= 3 children
