# Supplementary figures and images for: Serum IgE Reactivity Profiling in an Asthma Affected Cohort
Source: PLoS One. 2011 Aug 4;6(8):e22319. doi: 10.1371/journal.pone.0022319 (PMC3150333; doi:10.1371/journal.pone.0022319)

Figure S1. Visual representation of the reactivity patterns of cluster 3, 4 and 5.

**
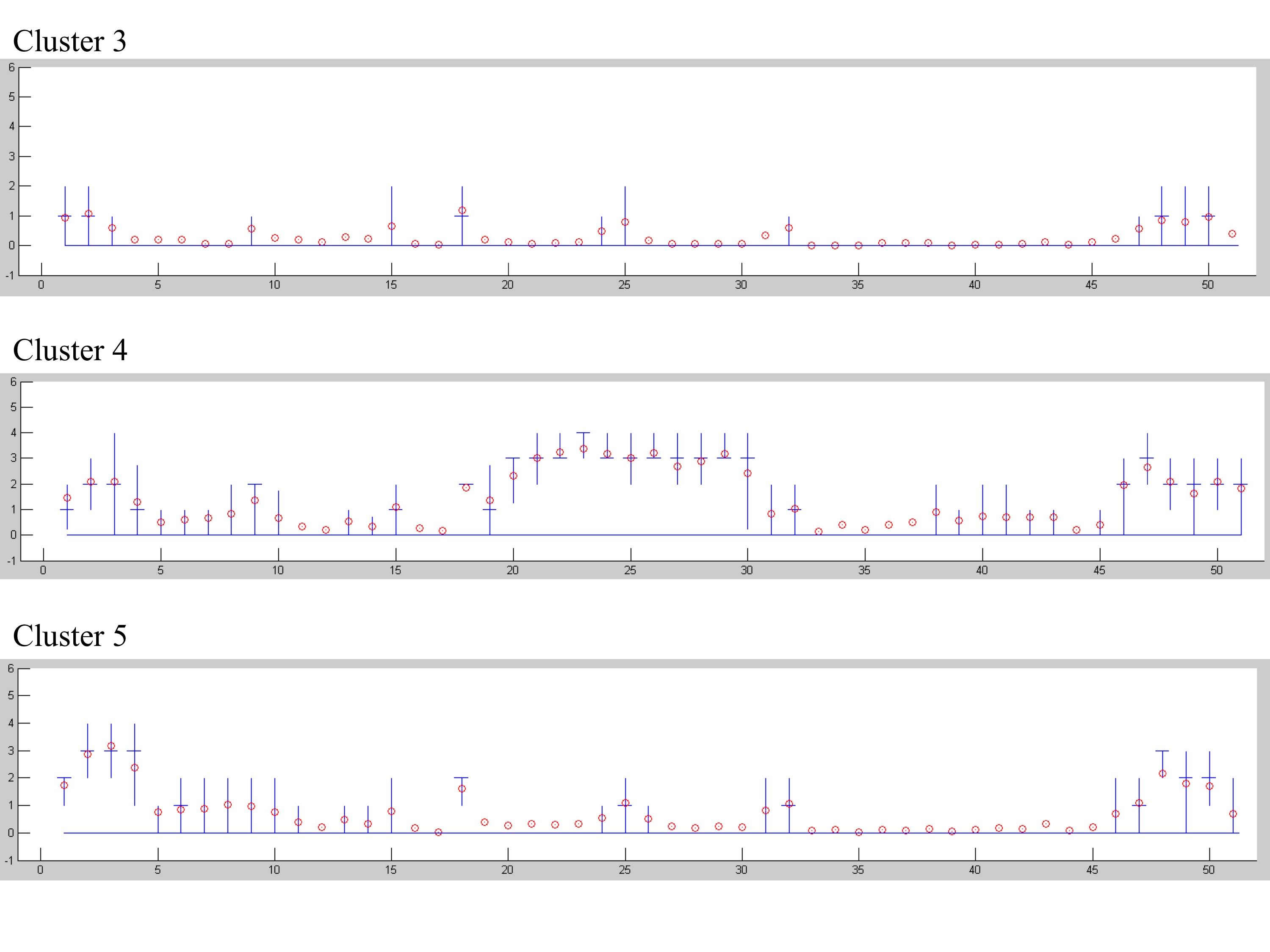
**

Supplement: Figure S1 — Visual representation of the reactivity patterns of cluster 3, 4 and 5. The numbers on the x axis correspond to the asthma relevant allergens (list on Table S6), the y axis shows the corresponding class score serum reactivity (0–5) in term of mean value red circles), the interquartile range (IQR- blue vertical lines) and the medians (horizontal lines of the IQR). (DOC) [file pone.0022319.s013.doc]
